# Association between Estrogen Levels and Temporomandibular Disorders: An Updated Systematic Review

**DOI:** 10.3390/ijms25189867

**Published:** 2024-09-12

**Authors:** Grzegorz Zieliński, Beata Pająk-Zielińska

**Affiliations:** 1 Department of Sports Medicine, Medical University of Lublin, 20-093 Lublin, Poland; 2 Interdisciplinary Scientific Group of Sports Medicine, Department of Sports Medicine, Medical University of Lublin, 20-093 Lublin, Poland

**Keywords:** temporomandibular joint, temporomandibular disorders, TMD, estrogen level, hormones, women, females, menopause, bruxism, orofacial pain

## Abstract

The aim of this systematic review is to evaluate the impact of estrogen levels on the occurrence of temporomandibular disorders (TMDs) in humans. Searches were conducted in the same databases as follows: PubMed, the Cochrane Collaboration database, and the Scopus database. In accordance with the MeSH database and previous work, the following keywords were used: ‘estrogens’ and ‘temporomandibular joint disorders’. Twelve studies were included in the review and were assessed for the quality of evidence. Estrogen levels are associated with pain modulation in the temporomandibular joint and the entire orofacial region. There is insufficient evidence to either confirm or refute the influence of estrogen on the occurrence of TMDs. The study was registered under the identifier: 10.17605/OSF.IO/BC7QF.

## 1. Introduction

The temporomandibular joint (TMJ) connects the mandible to the temporal bone of the skull and is a complex joint. It features an articular disc, an elastic, fibrous structure that separates the mandibular condyle from the articular fossa. The function of the TMJ is influenced by the following four primary muscles of mastication: the masseter, the temporalis, and the medial and lateral pterygoid muscles [1].

Temporomandibular disorders (TMDs) refer to pain and dysfunction of the TMJ and the surrounding tissues. Symptoms of TMDs include a limited range of motion and characteristic sounds during movement in the joint. It is worth emphasizing that TMDs are, in fact, a range of different disorders that can vary in etiology and symptoms (including internal derangement, osteoarthritis, myalgia) [2,3]. TMDs are the most common pain disorders within the oral and facial regions, and according to the latest meta-analysis from 2024, they affect 34% of the global population (26% in North America, 47% in South America, 33% in Asia, and 29% in Europe) [2].

Research indicates that TMDs are more prevalent in women [2,4,5,6]. The latest meta-analysis shows that the female-to-male ratio of TMDs prevalence is 1.26 in North America, 1.56 in South America, 1.26 in Asia, and 1.09 in Europe [2]. Other data suggest that women are approximately three times more likely to develop chronic TMDs [7].

Women exhibit greater stiffness and reduced relaxation following the deformation of aging TMJ discs [8]. Further studies indicate that the regenerative capacity of TMJ discs decreases with age in women [9]. Other studies have observed that adolescents with the occurrence of osteoarthritis of the temporomandibular joint showed premature skeletal maturation and a shorter predicted adult stature, particularly in female adolescents [10]. Robinson et al. observed gender differences in the ultrastructure of the extracellular matrix, which may affect the mechanical properties of the joint, likely regulated by sex hormones [7]. Fluctuations in estrogen levels during the reproductive and perimenopausal periods may predispose women to facial pain, while low estrogen levels after menopause may exacerbate TMJ degeneration and the loss of alveolar bone [7]. Since the discovery of estrogen receptors in TMJ in 1986, they have become a focus of intense research [11], particularly regarding the higher incidence of TMDs in women. These receptors are present in the cartilage tissues of TMJ and retrodiscal tissues [11].

Estrogen primarily acts on cells through estrogen receptors [12]. For instance, beta-estradiol exerts biological effects by binding to estrogen receptor alpha 1 (ESR1) present in the TMJ fibrocartilage [13], which increases the inflammatory cascade through the activation of specific matrix metalloproteinases (MMP-9 and MMP-13) in the TMJ [13,14]. ESR1 plays a crucial role in the development and progression of TMDs, and its expression has been mainly observed in certain TMJ discs [15]. Animal studies have shown that biomechanical stress and higher levels of estrogen lead to a reduction in TMJ cartilage thickness. Blocking ESR1 in animal models reduced the levels of MMP-9 and MMP-13 induced by 17β-estradiol [13].

**Figure 1:**
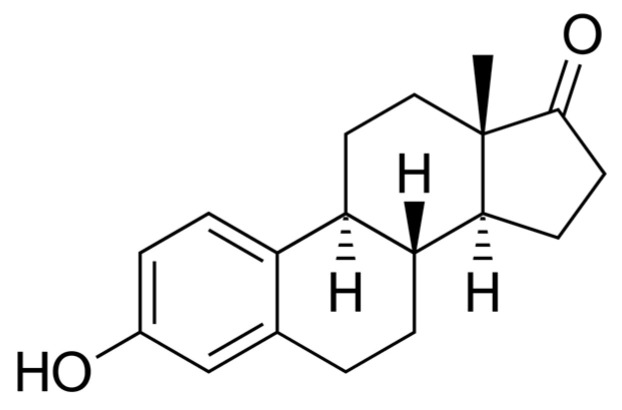
Estrone (E1)—chemical structure. Author: Edgar181, Public domain, via Wikimedia Commons.

**Figure 2:**
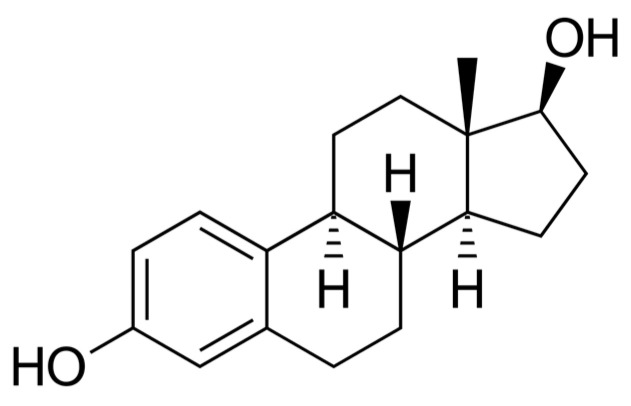
Estradiol (E2)—chemical structure. Author: NEUROtiker, Public domain, via Wikimedia Commons.

**Figure 3:**
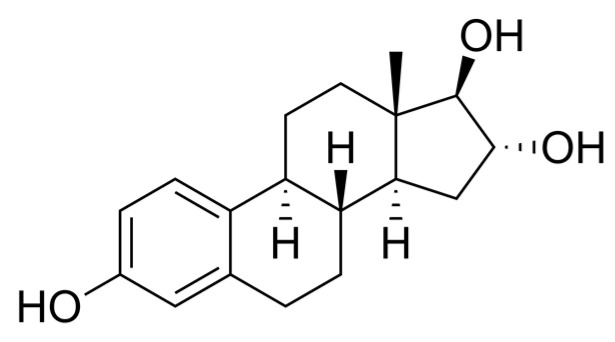
Estriol (E3)—chemical structure. Author: No machine-readable author provided. Ayacop assumed (based on copyright claims), Public domain, via Wikimedia Commons.

The breakthrough research step took place in 1929, when Adolf Butenandt and Edward Adelbert Doisy independently isolated and determined the structure of estrogen [16]. There are three main endogenous estrogens as follows: estrone (E1) (Figure 1), estradiol (E2) (Figure 2), and estriol (E3) (Figure 3) [17]. Estradiol, the most active estrogen during a woman’s reproductive years, plays a key role in many physiological functions and menstrual cycle processes.

According to the latest research, the serum estradiol (E2) concentrations are as follows: median 198 pmol/L (54 ng/L [18]) (5th–95th percentile) in the follicular phase, 757 pmol/L (206.21 ng/L [18]) during ovulation, and 412 pmol/L (112.23 ng/L [18]) in the luteal phase [19]. Other levels include, for example, estrone at the following concentrations: during the reproductive period at 17–200 ng/L and during the postmenopausal period at 7–40 ng/L. The estradiol levels are as follows: 30–120 ng/L (110–440 pmol/L) in the follicular phase, 130–370 ng/L (477–1358 pmol/L) during ovulation, 70–250 ng/L (257–917 pmol/L) in the luteal phase, and <10 ng/L during the postmenopausal period [20]. Very high levels of estradiol can be observed during pregnancy, for example, in the second trimester, reaching up to approximately 7000 pg/mL (7000 ng/L [18]) [21].

Various hypotheses have attempted to explain the influence of estrogen on the increased occurrence of TMDs in women. Berger et al. suggested that estrogen may modulate pain regulation mechanisms through its effects on estrogen receptors in both the peripheral and central nervous systems, potentially modifying pain signaling in both pro- and anti-nociceptive ways [11]. Bi et al. proposed that estrogen may increase hyperalgesia in the inflamed TMJ by lowering the pain threshold or by modulating the expression and threshold of sodium channel type 1.7 in the trigeminal ganglion [12].

Recent years have seen a systematic increase in studies investigating the impact of estrogen on TMDs [2,22,23]. The most recent systematic review on this topic was published in 2015 [11]. Given the growing number of dental studies and the lack of definitive answers in the previous reviews, a new review analyzing the years 2015–2024 has been undertaken. The aim of this systematic review is to evaluate the impact of estrogen levels on the occurrence of temporomandibular disorders in humans.

## 2. Methods

This systematic review follows the Preferred Reporting Items for Systematic Reviews and Meta-Analyses (PRISMA 2020 guidelines) [24] (Supplementary Material S1 and S2). The systematic review protocol was registered in the Open Science Framework (OSF) under the identifier: DOI 10.17605/OSF.IO/BC7QF [25].

The methodology developed by Berger et al. was replicated in this work [11]. Searches were conducted in the same databases as follows: PubMed, the Cochrane Collaboration database, and the Scopus database. The authors also performed manual searches using the Google search engine with keyword combinations to locate the gray literature sources [23]. In accordance with the MeSH database and previous work, the following keywords were used: ‘estrogens’ and ‘temporomandibular joint disorders’.

Studies published from 1 January 2015 to 30 June 2024 were included. The searching of the described databases took place from 29 July 2024 to 29 August 2024 [25]. Two researchers, G.Z. and B.P.-Z., independently conducted the review, and all discrepancies were resolved by consensus. Inclusion and exclusion criteria were developed according to PICO guidelines [26]–Table 1.

**Figure 4:**
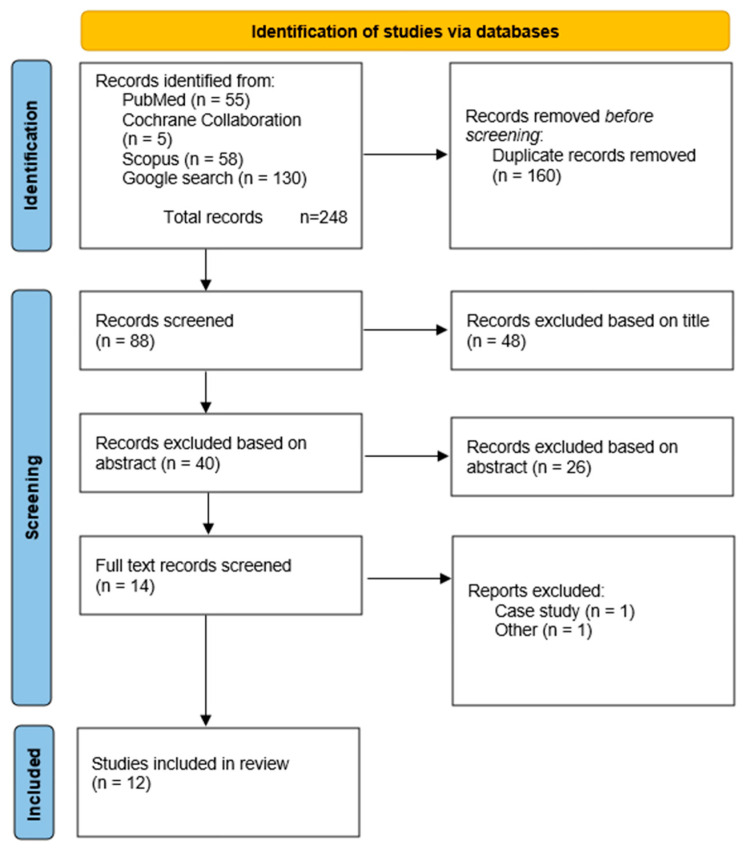
PRISMA flow diagram.

According to the previous publications, the titles were first reviewed, followed by the abstracts, and finally the full-text articles [2,23]. After removing the duplicates, 88 records were analyzed based on the titles, 40 based on the abstracts, and 12 based on the full-text articles. One case study was excluded [27], one paper was excluded because the full text could not be obtained. During the search period, the journal’s website was not operational, and a request was sent to the authors for access to the full text, but no response was received. Consequently, publications from the analysis were included [28] (Figure 4).

The assessment of the evidence collected in this systematic review was completed based on the questionnaire developed by Berger et al. [11]. The questionnaire created by the aforementioned author was based on the Cochrane Handbook for Systematic Reviews of Interventions [29], PRISMA [24], and the QUADAS tool [30]. Additionally, question Q5 was updated with the DC/TMD questionnaire, whereas it was previously based only on RDC/TMD [31]. The detailed questionnaire can be found in the Supplementary Material S3. The evaluation of the publications can be found in Table 2.

## 3. Results

### 3.1. Presentation and Evaluation of Qualified Studies

Ultimately, eight studies that met the inclusion and exclusion criteria were included in the analysis. These were works by Patil et al. (2015) [32]; Vilanova et al. (2015) [36]; Lora et al. (2016) [37]; Ribeiro-Dasilva et al. (2017) [38]; Ivković et al. (2018) [33]; Babouei et al. (2019) [39]; Fichera et al. (2020) [40]; Yuan et al. (2021) [34]; Yazici et al. (2021) [35]; Jedynak et al. (2021) [41]; Mursu et al. (2023) [42]; and Minervini et al. (2024) [43].

Patil et al. analyzed the estrogen levels in individuals with TMDs (based on the Fonseca Questionnaire) by collecting 5 mL of blood during the luteal phase. A total of 200 women aged 14–40 years were examined [32].

The remaining studies included in this analysis did not measure estrogen levels in a laboratory. Vilanova et al. studied 50 women, who were divided into the following two groups: menstrual cycle and oral contraceptive (25 each). The TMD symptoms were analyzed based on RDC/TMD. The average age was 25 years for the first group and 29 years for the second group [36].

The study by Lora et al., which included 284 patients, of whom 129 had TMDs as determined by RDC/TMD, had an average patient age of 56.7 years. The aim of this study was to investigate the prevalence of TMDs in postmenopausal women and its relationship with pain and hormone replacement therapy [37].

Ribeiro-Dasilva et al. examined 11 women with TMDs and 10 healthy women. The TMDs were assessed using RDC/TMD. The women were aged 18 to 35 years. Blood samples for analysis were collected during the follicular phase of the menstrual cycle [38].

Ivković et al. investigated the occurrence of TMDs in the following three groups of women: normally cycling women (Group 1), pregnant women (Group 2), and women in surgical menopause (Group 3). For these groups, they determined the estradiol levels. They measured estradiol from 2 mL of blood during the follicular phase in 125 women aged 20–40 years [33].

Babouei et al. conducted the study on 71 postmenopausal women and 69 non-menopausal women, aged 45–55 years. They used Helkimo’s clinical index [39].

The study conducted by Fichera et al. aimed to assess the presence of TMJ disorders in a cohort of pregnant women. They used the Helkimo index and included women aged 19 to 35 years. A total of 198 women participated in the study, including 108 subjects in the study group [40].

Yuan et al. studied the occurrence of idiopathic condylar resorption and disc displacement in both women and men and assessed their estradiol levels. They determined the changes based on MRI scans. The first group included 94 individuals (80 women, 14 men), and the second group included 324 individuals (259 women, 65 men). The estradiol levels were measured on day 2 and day 5 of menses from blood samples. The age range was 11 to 43 years [34].

Yazici et al. examined the following two groups of women: those with polycystic ovary syndrome (Group 45) and healthy women (Group 30). They assessed the estrogen levels and the occurrence of TMDs based on RDC/TMD. The first group comprised 45 women, while the second group included 30 women, with an age range of 20–40 years. The study was conducted during the early follicular and mid-luteal phases of menstruation, and the estrogen levels were measured from blood [35].

Jedynak et al. studied 126 women, 65 with hormonal disorders and 61 healthy women. They determined the occurrence of TMDs based on the DC/TMD and measured the estradiol levels from the blood. The age range of the examined women was 18–40 years [41].

Mursu et al. conducted a study aimed at examining the association between climacteric status and TMDs. In the study, women with a follicle-stimulating hormone level exceeding 25 IU/L and who had experienced amenorrhea for over 4 months were categorized as climacteric (n = 71). Those who did not meet these criteria were classified as preclimacteric (n = 656). The differences between the climacteric and preclimacteric groups were evaluated based on self-reported pain related to TMDs. DC/TMD was used to diagnose TMDs. The age of the participants was 46 years [42].

Another study was conducted by Minervini et al., who assessed whether certain factors related to pregnancy influence the prevalence or severity of TMDs. They investigated 32 pregnant women, including those aged 18 to 50 years. The researchers used the Graded Chronic Pain Scale, Jaw Functional Limitation Scale-20, and Oral Behaviors Checklist to evaluate their TMDs [43].

According to the classification developed by Berger et al. [11], the studies by Vilanova et al. [36], Babouei et al. [39], and Minervini et al. [43] were classified as having poor value of evidence. Patil et al. [32], Lora et al. [37], Ribeiro-Dasilva et al. [38], Fichera et al. [40], Yuan et al. [34], Yazici et al. [35], Jedynak et al. [41], and Mursu et al. [42] were classified as having moderate evidence. The study by Ivković et al. [33] was classified as having high evidence (Table 3).

During the analysis of the available literature, it was observed that there is significant heterogeneity in the methods of collecting material for estrogen analysis. The authors in the analyzed studies collected blood but in varying amounts and at different phases of the cycle. They also examined different age ranges and presented various measurement units. Undoubtedly, this significantly complicates the uniform analysis of the collected data. Even after converting units to the same measurement (ng/L) [18], it is not possible to conduct a systematic comparison due to differences in the patient populations and analytical methods. Undoubtedly, it is important to highlight the intriguing finding from the study by Patil et al. [32] that an increase in estrogen levels is associated with variations in the severity of TMDs. However, this observation should be verified in future research. The data are presented in Table 2.

### 3.2. Synthesis of Systematic Reviews

LeResche et al. [44,45,46] suggested a positive association between the pain related to TMDs and the hormone levels. Mursu et al. [42] obtained similar results. Dao et al. [47] recognized estrogen’s role in the etiopathology of inflammatory conditions. In contrast, studies by Hatch et al. [48] and Sherman et al. [49] questioned the impact of estrogens on pain responses. Ribeiro-Dasilva et al. suggested that an estrogen-induced hyperinflammatory phenotype in women with TMDs may, at least in part, contribute to heightened clinical pain [38]. Nekora-Azak et al. [50] did not observe a significant difference in TMDs signs and symptoms between postmenopausal women undergoing hormonal therapy and those not receiving postmenopausal hormones. Babouei et al. identified TMDs as one of the issues related to menopause [39].

Landi et al. [51,52] discussed a direct connection between the estrogen levels and the TMDs, similar to Patil et al. [32], who noted that the estrogen levels influence the severity of the TMD symptoms. Ivković et al. [33] also observed that TMD signs and symptoms may be modulated and sustained by estrogen levels, a view supported by the research of Jedynak et al. [41], Vilanova et al. [36], and Minervini et al. (2024) [43]. According to Fichera et al., pregnant females may be more susceptible to TMDs. They explain this by an increase in the level of estrogen hormones [40].

Conversely, studies by Lora et al. [37], Yuan et al. [34], and Yazici et al. [35] present opposing findings.

**Figure 5:**
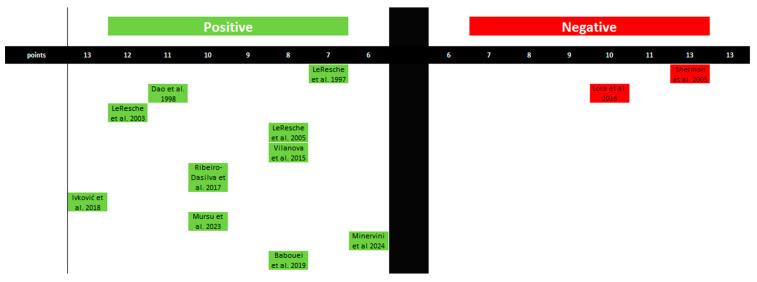
Graphical representation of studies linking estrogen levels to pain levels in the orofacial region. The figure was created based on the findings in Table 4.

A synthesis and graphical presentation of studies linking estrogen levels to the exacerbation of pain in the TMJ and the orofacial region have been conducted. In this case, 10 studies have confirmed the influence of estrogen on the occurrence of increased pain. Two studies stand in opposition. In this case, the impact of estrogen on pain levels in the orofacial and TMJ regions can be confirmed (Figure 5 and Table 4).

**Figure 6:**
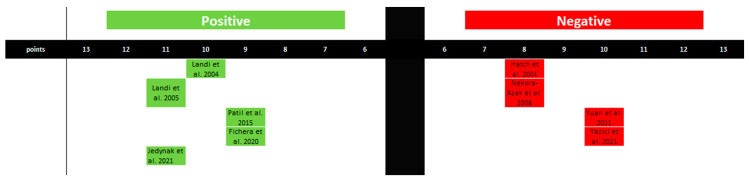
Graphical representation of studies linking estrogen levels to the occurrence of TMJ disorders. The figure was created based on the findings in Table 4.

Regarding the influence of estrogen on the development of TMDs, five studies confirm this association, while four present a negative relationship. This, however, highlights discrepancies in the results and the inability to conclusively establish a correlation between estrogen levels and the occurrence of TMDs (Figure 6 and Table 4).

## 4. Discussion

The primary objective of the study was to evaluate the impact of estrogen levels on the occurrence of temporomandibular disorders in humans. The objective was achieved; however, when considering the results in conjunction with the work of Berger et al. [11], the following division emerged: Up to 6 studies do not confirm the association, while 15 support it (Table 4). During the synthesis of both systematic reviews, the following two trends were observed: one regarding the impact of estrogens on larger pain conditions (Figure 4), and the other concerning the development of TMDs (Figure 5). Analyzing the collected evidence, the first trend appears to be highly plausible, while the second remains a hypothesis.

For instance, researchers have presented the impact of estrogens on pain levels using animal models, for example, Zhang et al. indicated that these results suggest a mild, localized inflammatory state in the TMJ during periods of elevated estrogen levels was sufficient to increase the peripheral drive for movement-induced hyperalgesia [53]. Hornung et al. noted that a lower dose of progesterone might effectively reduce the recurrence of estrogen-evoked inflammatory mechanical allodynia in the TMJ [54]. Recent research (2024) in the therapeutic context by Kroeff et al. demonstrated that hormone replacement therapy did not alleviate orofacial pain in ovariectomized rats. However, there was a decrease in brainstem TNF-ɑ levels in the animals subjected to both models, which was partially reversed by estrogen hormone replacement therapy [55].

Chen et al., in their 2021 review, observed that estrogen and estrogen receptors (ERs) play a role in modulating pain across various commonly utilized animal models, including those for peripheral and central neuropathic pain, inflammatory pain, and hyperalgesia priming. While different signaling pathways involving ERs have been identified, the exact mechanisms by which ERs influence pain remain largely unclear. The existing animal models do not replicate all pain types or fully capture the effects of ERs on pain modulation [56]. Kroeffet al. point out that pain perception is affected by many factors, such as the type of pain, location, estrogen levels, and gender [55].

In the context of the relationship between estrogen and the development of TMDs, there are currently numerous studies using animal models, for example, Doetzer et al. demonstrated no significant difference between the condyle fracture, anterior disk displacement with reduction, and anterior disk displacement without reduction groups concerning age and the expression of estrogen receptor alpha 1, as observed through immunohistochemical examination [14]. This is supported by the studies presented earlier [12]. Robinson et al. provided evidence that sex differences in decreased occlusal loading-induced inhibition of collagen type II expression do not appear to be mediated by the differences in estradiol levels between the male and female mice [57]. Xue et al. observed that estrogen-sensitized synoviocytes in female rats may contribute to gender differences in the incidence and progression of temporomandibular joint osteoarthritis [58]. Zhao et al. demonstrated that estrogen-related receptor γ is a downstream transcription factor of extracellular signal-regulated kinase 1/2, and its upregulation leads to extracellular matrix degradation and angiogenesis in temporomandibular joint osteoarthritis. This study identified a common factor between inflammation and vascularization in osteoarthritis [59]. Ye et al. reached similar conclusions, showing that high estrogen levels play a destructive role in condylar cartilage but a protective role in subchondral bone during the early stages of temporomandibular joint osteoarthritis. These dual and divergent effects should be seriously considered in future osteoarthritis therapies [60]. In a clinical context, studies on estrogen-induced TMJ changes were conducted by Abdrabuh et al. They observed that estrogen deficiency degraded some TMJ structures, with only minimal recovery following estrogen replacement therapy [61]. Despite the numerous studies on animal models confirming the relationship between the estrogen levels and the TMDs, the current research on humans is not conclusive (Figure 6 and Table 4).

The above information, in our opinion, confirms the observation that estrogen levels will affect modulation within the TMJ, but it is impossible to say whether the level of estrogen will be associated with the occurrence of TMDs.

In the context of examining the potential impact, or lack thereof, of estrogen levels on TMDs, it is valuable to review the latest epidemiological data. As mentioned in the introduction, the most recent meta-analysis indicates that the female-to-male ratio of TMDs prevalence is 1.26 in North America, 1.56 in South America, 1.26 in Asia, and 1.09 in Europe [2]. However, according to the authors’ results, the differences between groups yielded a *p*-value of 0.286. The authors concluded that while there were regional differences in the TMD diagnosis based on gender, these differences may not be statistically significant [2].

The above epidemiological data, along with the presented human studies, might suggest that estrogen levels do not directly influence the occurrence of TMDs symptoms. However, observable differences in the prevalence of TMDs between women and men over the years persist. Despite the lack of statistically significant differences reported in the latest meta-analysis, the female-to-male ratios vary. Other factors, such as bruxism, should be considered in this context.

The most recent meta-analysis (2023) by Mortazavi et al. indicated a positive association between bruxism and TMDs. The authors noted that the presence of bruxism increases the likelihood of developing TMDs in the future [62]. In light of these findings, it is also pertinent to consider the latest epidemiological data on bruxism. Bruxism, whether awake or during sleep, is more prevalent among women of all ages. For instance, awake bruxism occurs in 18% of adult women compared to 9% of men, while sleep bruxism affects 15% of adult women and 8% of men. Additionally, age was found to be a significant factor influencing the occurrence of sleep bruxism among women (data for men were not observed) [23]. In earlier studies, it was observed that women clench their teeth 22% more often compared to men [63]. Subsequent studies, along with the latest 2024 meta-analysis [23], confirm that various forms of bruxism are more common in women [64].

These observations provide a hypothetical explanation for the higher prevalence of TMDs among women, which warrants further investigation in future studies.

This study has several limitations, primarily due to the different methodologies and analyses used, which prevented the conduct of a meta-analysis. Only six studies in the current review conducted laboratory analyses of estrogen levels, and only four provided numerical data in the text (Table 2). Another limitation is that researchers often performed analyses at various phases of the menstrual cycle, which complicates the synthesis of the literature. This significantly hinders, for example, the precise calculation of estrogen levels responsible for pain modulation or confirming the impact on the TMDs. Additionally, this analysis was conducted by authors different from those of the initial study by Berger et al; although the criteria and evaluation described by the cited author were used, there may hypothetically be an associated error. However, the authors of the current analysis assert that they made every effort to avoid such errors. 

## 5. Conclusions

Thus, the conclusions of this review are as follows:

- Estrogen levels are associated with pain modulation in the temporomandibular joint and the entire orofacial region.
- There is insufficient evidence to either confirm or refute the influence of estrogen on the occurrence of TMDs.

## Figures and Tables

**Table 1:** PICO Summary of Inclusion and Exclusion Criteria.

	Inclusion	Exclusion
Population		
	Women of various ages suffering from TMDs.	Men of various ages suffering from TMDs.
Intervention		
	Estrogen levels, e.g., endogenous estrogen levels, estrogen supplementation, hormone replacement therapy.	
Comparison		
	Control group: women with normal estrogen levels, no estrogen supplementation, placebo, or no TMDs.	
Outcome		
	Impact on the severity of TMDs symptoms, e.g., pain, limited mobility, sounds during movement in the joint (clicking), overall quality of life.	
Study Design		
		Narrative Reviews Systematic Articles Meta-analyses Expert Opinions Language other than English

Table created on the basis of researches [2,11,23,25].

**Table 2:** The table presents the most important information regarding studies that measured estrogen levels.

	Number of People and Group Information		Estrogen Levels	Measurement Unit in Researches	Converted Units ng/L
Patil et al. [32]	Severe cases of TMDs	59	444.20	pg/mL	444.20
	Moderate cases of TMDs	66	264.50		264.50
	Mild cases of TMDs	75	199.80		199.80
	Total	200	302.10		302.10
Ivković et al. [33]	Normally cycling women	TMDs n–28 (56%) Total n–50	0.10	ng/mL	100
	Pregnant women	TMDs n–23 (51%) Total n–45	14.67		14,670
	Women in surgical menopause	TMDs n–13 (43%) Total n–30	0.02		20
Yuan et al. [34]	Idiopathic condylar resorption	80	36.93	pg/mL	36.93
	Disc displacement	259	34.36		34.36
Yazici et al. [35]	Control group	TMDs n–2 (7%) Total n–30	44.3	ng/mL	44,300
	Polycystic ovary syndrome	TMDs n–23 (51%) Total n–45	47.6		47,600

**Table 3:** Assessment of evidence found in publications.

	Q1	Q2	Q3	Q4	Q5	Q6	Q7	Total
Patil et al. 2015 [32]	2	0	1	1	1	2	2	9
Vilanova et al. 2015 [36]	1	2	1	0	2	1	1	8
Lora et al. 2016 [37]	2	2	1	0	2	1	2	10
Ribeiro-Dasilva et al. 2017 [38]	1	2	1	0	2	2	2	10
Ivković et al. 2018 [33]	2	2	1	2	2	2	2	13
Babouei et al. 2019 [39]	1	2	1	0	2	1	1	8
Fichera et al. 2020 [40]	2	2	1	0	1	1	2	9
Yuan et al. 2021 [34]	2	0	2	1	1	2	2	10
Yazici et al. 2021 [35]	1	2	1	0	2	2	2	10
Jedynak et al. 2021 [41]	2	2	1	0	2	2	2	11
Mursu et al. 2023 [42]	2	2	1	0	2	1	2	10
Minervini et al. 2024 [43]	1	0	1	0	1	1	2	6

**Table 4:** Synthesis of obtained results based on Berger’s [11] analyses and original work.

Author	Conclusion	Evaluation	Summary of Estrogen’s Effect on TMDs
LeResche et al., 1997 [44] *	“These results suggest that female reproductive hormones may play an etiologic role in orofacial pain”.	7	Positive
Dao et al., 1998 [47] *	“(…)Evidence supporting the link between estrogen, nitric oxide, and inflammatory processes is presented”.	11	Positive
Hatch et al., 2001 [48] *	“Estrogen replacement therapy does not place women at increased risk of developing TMDs”.	8	Negative
LeResche et al., 2003 [45] *	“These results suggest that TMD pain in women is highest at times of lowest estrogen, but rapid estrogen change may also be associated with increased pain”.	12	Positive
Landi et al., 2004 [51] *	“The results of this study suggest that high serum estrogens levels might be implicated in the physiopathology of temporomandibular joint disorders, since subjects with these pathologies showed significantly higher serum levels with respect to a group of healthy controls”.	10	Positive
LeResche et al., 2005 [46] *	“Musculoskeletal orofacial pain and related symptoms appear to improve over the course of pregnancy. This improvement occurs in the presence of increased joint laxity and is not paralleled by improvements in psychological distress. Thus, it was concluded that the improvement in pain is most likely associated with the dramatic hormonal changes occurring during pregnancy”.	8	Positive
Landi et al., 2005 [52] *	“Considering the multifactorial etiology of TMD and the hypothesis that some joint tissues (e.g., bone, cartilage, collagen, proteins) could be a target for sexual hormones, these data suggest that high serum estrogen levels might be implicated in the physiopathology of TMD”.	11	Positive
Sherman et al., 2005 [49] *	“Phase-related differences in experimental pain response were not strong and were more often found for experimental stimuli with greater clinical relevance (ie, palpation pain) compared with an ischemic pain task”.	12	Negative
Nekora-Azak et al., 2008 [50] *	“There was no significant difference found in the signs and symptoms of TMD between postmenopausal women using hormone therapy and those not using postmenopausal hormones. There was no association between the use of postmenopausal hormones and the signs and symptoms of TMD in this study”.	8	Negative
Patil et al., 2015 [32]	“Increasing serum levels of estrogen and progesterone with increasing grade of severity of TMD suggest a role of these hormones as etiological factors for TMD”.	9	Positive
Vilanova et al., 2015 [36]	“Hormonal fluctuations intensify pain in women with symptomatic TMD without impairing mastication”.	8	Positive
Lora et al., 2016 [37]	“There was a similar percentage of TMD and non TMD patients; moreover, the use of exogenous hormones was no associated with TMD, suggesting that there is no influence on the pain threshold”.	10	Negative
Ribeiro-Dasilva et al., 2017 [38]	“These data suggest that an estrogen-induced hyperinflammatory phenotype in women with TMD may at least in part contribute to heightened clinical pain, perhaps via central sensitization”.	10	Positive
Ivković et al., 2018 [33]	“TMD signs and symptoms may be modulated and sustained by estrogen levels. (…) Regulating the level and variations of estrogen across the hormonal cycle could be a promising approach for the treatment of TMD”	13	Positive
Babouei et al., 2019 [39]	“In the results of this study, it was found that the indices of pain in (TMJ, decuple masseter muscles and on mandibular movements) in women are most severe when estrogen levels are at a minimum; it was also found that decreased estrogen secretion in postmenopausal phase (…) caused increased inflammation and reduced development and progress of TMJ tissue components which is the reason for increased incidence and severity of all five symptoms of TMD based on the Helkimo’s clinical index (…)”.	8	Positive
Fichera et al., 2020 [40]	“Female subjects in pregnancy status could be more susceptible to TMD due to a physiological increment of estrogenic hormones levels. However, further studies are needed to better understand the role of TMD during pregnancy”.	9	Positive
Yuan et al., 2021 [34]	“(…) there were no differences in serum E2 levels or the levels of other sex hormones between female ICR and DD patients”.	10	Negative
Yazici et al., 2021 [35]	“In the comparison of estrogen levels, no significant difference was found between groups. -Information from the results section”.	10	Negative
Jedynak et al., 2021 [41]	“(…) In women with TMD symptoms, their medical history should be extended to include the diagnosis of female hormone disorders”.	11	Positive
Mursu et al., 2023 [42]	“In conclusion, among females at the age of 46 years, climacterium seems to be associated with TMD by increasing pain on palpation in TMJs and subjective symptoms and clinical signs indicating degenerative changes in TMJs when using DC/TMD”.	10	Positive
Minervini et al., 2024 [43]	“In conclusion, our study identified significant associations between psychosomatic and psychological symptoms with variables like age and pregnancy trimester in pregnant women”.	6	Positive

*—Works taken from Berger’s analysis.

## Data Availability

All the data is provided in the Supplementary Materials and through the registration link. https://doi.org/10.17605/OSF.IO/BC7QF.

## Supplementary Materials

The following supporting information can be downloaded at: https://www.mdpi.com/article/10.3390/ijms25189867/s1.
