# Supplementary material for: Association between Estrogen Levels and Temporomandibular Disorders: An Updated Systematic Review
Source: Int J Mol Sci. 2024 Sep 12;25(18):9867. doi: 10.3390/ijms25189867 (PMC11432328; doi:10.3390/ijms25189867)
Supplement: Supplementary file 1 [file ijms-25-09867-s001.zip › Supplementary Material S3 Adaptation of the evaluation questionnaire..pdf]

Systematic Review

Association Between Estrogen Levels and Temporomandibular Disorders: An Updated Systematic Review

Grzegorz Zieliński [1,*], Beata Pająk-Zielińska [2],

[1] Department of Sports Medicine, Medical University of Lublin, Poland;
[2] Interdisciplinary Scientific Group of Sports Medicine, Department of Sports Medicine, Medical University of Lublin, Poland;
[*] Correspondence: Grzegorz Zieliński, grzegorz.zielinski@umlub.pl

The following table and assessment criteria were originally developed by Berger et al. [1]. We made modifications to question Q5 according to the guidelines [2].

Table S3. Protocol for assessing studies qualified for meta-analysis.

	Questions	Scoring	Changes
**Q1**	Sample size	0-9 – 0 pts<br>10-99 – 1 pt<br>>100 – 2 pts	
**Q2**	Control group	none – 0 pts<br>present – 2 pts	
**Q3**	Inclusion criteria	none – 0 pts<br>present, not restricted to one form of TMD – 1 pt<br>restricted to one form of TMD – 2 pts	
**Q4**	Exclusion criteria	none – 0 pts<br>concomitant pain disorders OR other medications use – 1 pt<br>concomitant pain disorders AND other medications use – 2 pts	
**Q5**	Use of standardized examination protocol	none – 0 pts<br>other protocols – 1 pt<br>RDC/TMD and DC/TMD – 2 pts	Addition of 'DC/TMD' protocol
**Q6**	Estrogen level assessment	none – 0 pts<br>assessment based on the phase of the menstrual cycle – 1 pt<br>measured in blood or saliva samples – 2 pts	
**Q7**	Conflict of interest	present – 0 pts<br>no data – 1 pt<br>none – 2 pts	
**Scores 0-8 were rated as the poor value of evidence;<br>Scores 9-11 were rated as the moderate value of evidence;<br>Scores 12-14 were rated as the high value of evidence.**			

References

1. Berger, M.; Szalewski, L.; Bakalczuk, M.; Bakalczuk, G.; Bakalczuk, S.; Szkutnik, J. Association between Estrogen Levels and Temporomandibular Disorders: A Systematic Literature Review. *Menopause Rev. Menopauzalny* **2015**, *14*, 260–270, doi:10.5114/pm.2015.56538.
2. Schiffman, E.; Ohrbach, R.; Truelove, E.; Look, J.; Anderson, G.; Goulet, J.-P.; List, T.; Svensson, P.; Gonzalez, Y.; Lobbezoo, F.; et al. Diagnostic Criteria for Temporomandibular Disorders (DC/TMD) for Clinical and Research Applications: Recommendations of the International RDC/TMD Consortium Network* and Orofacial Pain Special Interest Group†. *J. Oral Facial Pain Headache* **2014**, *28*, 6–27, doi:10.11607/jop.1151.
